# Uncommon mandibular osteomyelitis in a cat caused by *Nocardia africana*

**DOI:** 10.1186/1746-6148-8-239

**Published:** 2012-12-06

**Authors:** Marconi Rodrigues de Farias, Juliana Werner, Márcio Garcia Ribeiro, Sabrina Marin Rodigheri, Carolina Zaghi Cavalcante, Kung Darh Chi, Larissa Anuska Zeni Condas, Tohru Gonoi, Tetsuhiro Matsuzama, Katsukiyo Yazama

**Affiliations:** 1 Companion Veterinary Hospital, College of Veterinary Medicine, Pontifícia Universidade Católica, São José dos Pinhais, Parana, Brazil; 2 Department of Veterinary Hygiene and Public Health, School of Veterinary Medicine and Animal Sciences, Univ. Estadual Paulista-UNESP, Botucatu, Sao Paulo, Brazil; 3 Companion Animal Surgical Practice, FEPAR, Curitiba, Parana, Brazil; 4 Medical Mycology Research Center, Chiba University, Chiba, Japan; 5 Infectious Diseases of Domestic Animals, Department of Veterinary Hygiene and Public Health, School of Veterinary Medicine and Animal Sciences, Univ. Estadual Paulista-UNESP, Code 18618-970, POBox 560, Botucatu, Sao Paulo, Brazil

**Keywords:** Cat, *Nocardia africana*, Feline nocardiosis, Osteomyelitis

## Abstract

**Background:**

Nocardiosis is an unusual infection in companion animals characterized by suppurative to pyogranulomatous lesions, localized or disseminated. Cutaneous-subcutaneous, pulmonary and systemic signs are observed in feline nocardiosis. However, osteomyelitis is a rare clinical manifestation in cats. *Nocardia cyriacigeorgica* (formerly *N. asteroides* sensu stricto), *Nocardia brasiliensis, Nocardia otitidiscaviarum*, and *Nocardia nova* are the most common pathogenic species identified in cats, based on recent molecular classification (16S rRNA gene). The present report is, to our knowledge, the first case of mandibular osteomyelitis in a cat caused by *Nocardia africana*, diagnosed based upon a combination of methods, including molecular techniques.

**Case presentation:**

A one-year-old non-neutered female cat, raised in a rural area, was admitted to the Companion Animal Hospital-PUCPR, São José dos Pinhais, State of Paraná, Brazil, with a history a progressive facial lesion, difficulty apprehending food, loss of appetite, apathy and emaciation. Clinical examination showed fever, submandibular lymphadenitis, and a painless, 8 cm diameter mass, which was irregularly-shaped, of firm consistency, and located in the region of the left mandible. The skin around the lesion was friable, with diffuse inflammation (cellulitis), multiple draining sinuses, and exudation of serosanguinous material containing whitish “sulfur” granules.

Diagnosis was based initially in clinical signs, microbiological culture, cytological, and histopathological findings, and radiographic images. Molecular sequencing of 16S rRNA of isolate allowed diagnosis of *Nocardia africana*. Despite supportive care and antimicrobial therapy based on in vitro susceptibility testing the animal died.

**Conclusion:**

The present report describes a rare clinical case of feline osteomyelitis caused by *Nocardia africana*, diagnosed based upon a combination of clinical signs, microbiological culture, cytological and histopathological findings, radiographic images, and molecular methods. The use of modern molecular techniques constitutes a quick and reliable method for *Nocardia* species identification, and may contribute to identification to new species of *Nocardia* that are virulent in cats.

## Background

Nocardiosis is an opportunistic disease for humans and domestic animals, caused by aerobic actinomycetes from the genus *Nocardia*. These organisms are ubiquitous, soil saprophytes, and are found in organic material, water and plants.

In companion animals, nocardial infection is considered rare, and are characterized by suppurative to pyogranulomatous lesions, localized or disseminated [1].

Routine diagnosis in companion animals is based on microbiological culture, and phenotypic characterization [2]. Currently, species of the genus *Nocardia* were reclassified using molecular methods. *Nocardia cyriacigeorgica* (formerly *N. asteroides* sensu stricto), *Nocardia brasiliensis, Nocardia otitidiscaviarum*, and *Nocardia nova* complex are the most pathogenic species involved in feline nocardiosis [3].

The transmission of feline nocardiosis is intimately related with the inhalation of soil organisms, and traumatic inoculation through wounds. Immunosuppressive diseases, such as immunodeficiency and leukemia in cats have been reported concurrently with feline nocardiosis [4]. Clinically, feline nocardiosis is characterized by cutaneous-subcutaneous lesions (abscesses, mycetoma, cellulitis), with draining sinuses, and less frequently by pulmonary and systemic forms, such as peritonitis and encephalitis [3].

Currently, human nocardiosis has emerged as an increasingly common opportunistic pathogen among immunosuppressed patients, particularly those infected by acquired immune deficiency syndrome–AIDS, other diseases (tuberculosis, lymphoma, lymphosarcoma, cirrhosis, diabetes), or debilitating conditions (organ transplantation, alcoholism, or prolonged use of corticosteroids) [3,5].

The present report describes a rare clinical case of osteomyelitis in a cat caused by *Nocardia africana.*

## Case presentation

**Figure 1:**
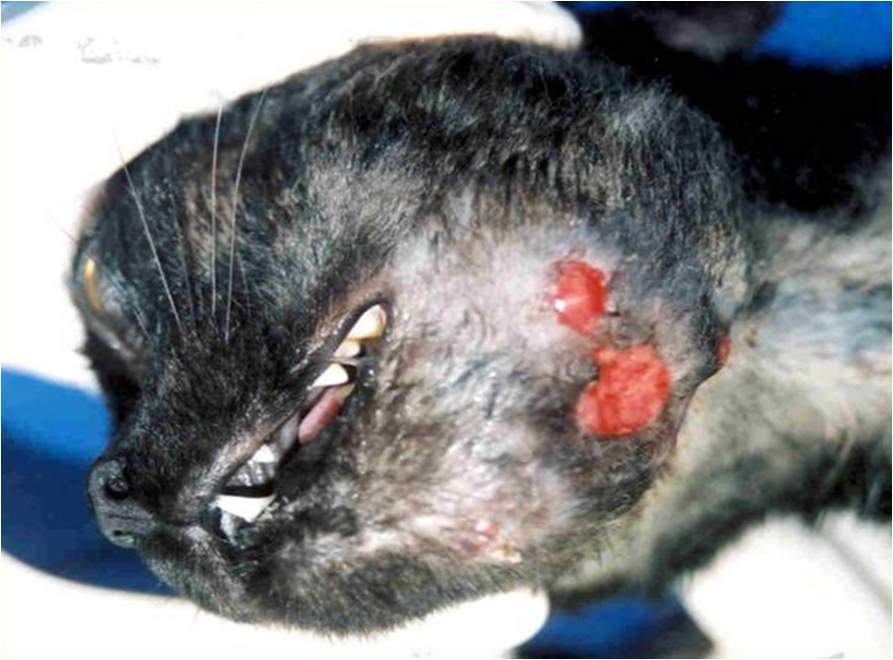
**Facial deformity and cellulites in the left mandibular region in a one-year-old female cat, caused by *Nocardia africana.***

A one-year-old non-neutered female cat, raised in a rural area, was admitted to the Companion Animal Hospital-PUCPR, São José dos Pinhais, State of Paraná, Brazil. The cat had a history a progressive facial lesion, difficulty apprehending food, loss of appetite, apathy and emaciation for approximately five months. The animal had no history of lesions in the mandibular region or mouth. Clinical examination showed fever, submandibular lymphadenitis and a painless, 8 cm diameter mass, which was irregular-shaped, of firm consistency, and located in the region of the left mandible (Figure 1). The skin around the lesion was friable, with diffuse inflammation (cellulitis), multiple draining sinuses, and exudation of serosanguinous material containing whitish “sulfur” granules. A blood sample for a complete blood count (CBC) was taken. Hematologic abnormalities included leukocytosis (21,400/μL, reference interval [RI] 6,000-17,000/μL), neutrophilia (18,200/μL, RI 3,000-11,500/μL), monocytosis (2,640/μL, RI 200–1,400/μL), and normocytic, normochromic, non-regenerative anemia (RBC 1.2x10^6^/μL, RI 5.5-8.5 x10^6^/μL; HCT 18%, RI 37-55%). Biochemical tests for renal and hepatic function were normal. Serological tests for feline immunodeficiency virus (FIV) and feline leukemia virus (FeLV) were negative (Snap FIV Antibody/FeLV Antigen Combo Test – IDEXX Laboratories, USA).

**Figure 2:**
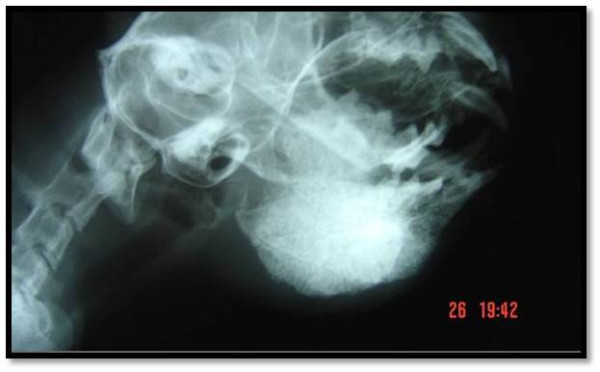
**Facial radiograph of the mandibular region in a cat with osteomyelitis caused by *Nocardia africana.*** Note severe osteolysis and bony proliferation in the mandible.

**Figure 3:**
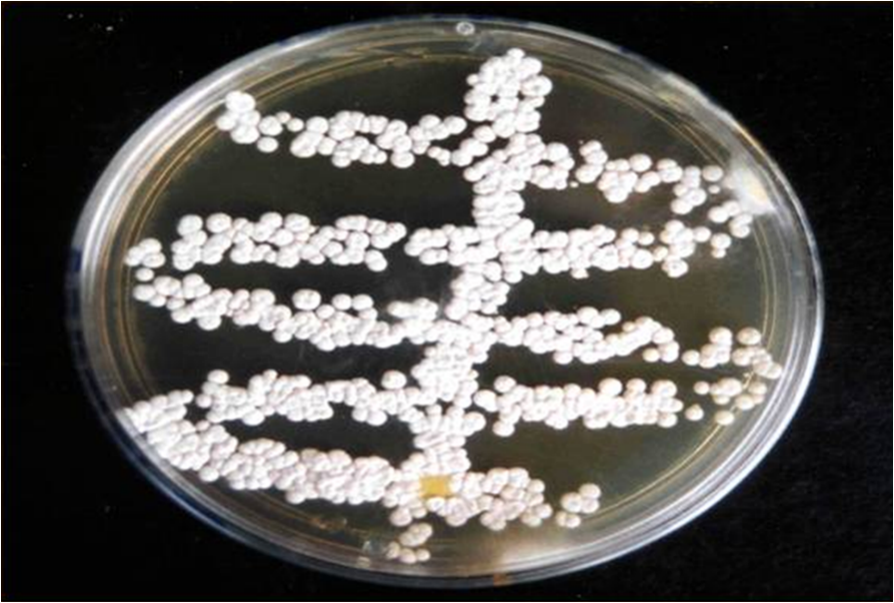
**Whitish-orange, circular, convex, dry, with powdery surface colonies aspect of *Nocardia africana *in Sabouraud agar after 72 h of incubation, isolated from a cat with mandibular osteomyelitis.**

Radiography of the mandible showed intense bone proliferation and osteolysis (Figure 2). Initially, fine needle aspiration was performed in the lesion [5] and sample was submitted simultaneously for cytological analysis and microbiological culture. Sample obtained by fine needle aspiration was stained with Gram and Panoptic stains, and showed numerous gram-positive, filamentous branching organisms. Furthermore, biopsy of lesion was taken. This fragment was stained with haemathoxilin-eosin and revealed a central region of necrosis and suppuration containing filamentous organisms, surrounded by neutrophils, macrophages, epithelioid cells and fibroblasts. Specimens were cultured on sheep blood agar and Sabouraud agar, incubated aerobically at 37°C for 72–96 h. After 72 h, numerous whitish-orange, circular, convex, dry, with powdery surface colonies were observed in sheep blood agar and Sabouraud agar (Figure 3). From these colonies, gram-positive, partially acid-fast (modified Ziehl-Neelsen), filamentous microorganisms were observed, suggestive of the genus *Nocardia*[2]*.*

Classical biochemical tests based on casein, xanthine, hypoxanthine, and tyrosine assimilation were carried out, as well use of other substrates in a commercial kit (API 20C AUX strips, BioMeriex, Hazelwood, MO.) [6]. These tests allowed the preliminary phenotypic classification of isolate as *Nocardia nova* complex [7].

Molecular analysis of the isolate was based on a subunit 16S rDNA gene sequence. Genomic DNA for sequencing was performed using guanidinium thiocyanate method [8]. DNA sequences were determined with an automatic sequence analyzer (Abi Prismtm 3130; Applied Biosystems), using the same primers and dye terminator cycle sequencing kit (Applied Biosystems). Nearly complete 16S rRNA gene sequence of approximately 1400 bases was obtained. The sequence was compared against GenBank database using BLAST, and sequences of related *Nocardia* type strains were retrieved from the database. Phylogenetic trees were constructed using the neighbor-joining method [9]. Topology of the trees was evaluated using bootstrap analysis of the sequence data using Mega4 software [10]. The sequencing analysis of the 16S rDNA identified the organism as *N. africana*, based on 99% sequence similarity with the reference sequence. The obtained sequence was submitted to GenBank/JJBJ/EMBL (accession No. AB636472 for the strain IFM 11168).

*In vitro* antimicrobial susceptibility test using the disk diffusion method [11] showed that the isolate was susceptible to trimethoprim-sulfonamide, amikacin, gentamicin, enrofloxacin, ceftiofur and amoxicillin/potassium clavulanate.

Trimethoprim-sulfonamide (30 mg/kg, PO, 12 h, for 21 days), combined with fluid therapy, debridement and infusion of topical (1%) iodine solution into the lesion were used initially in therapy. However, the clinical picture worsened, and treatment was changed to amikacin (10 mg/kg, IV, 12 h) and ceftiofur (4,4 mg/kg, SC, 12 h). After eight weeks of treatment, the animal still presented fever, weight loss, apathy, anorexia, and progressive worsening of body condition, which lead to death. *Post-mortem* examination showed abscess formation in the submandibular lymph nodes, severe osteolysis in the left mandibular ramus, and signs of periodontitis in mandibular region of lesion. There were no lesions in other organs. Purulent secretions from the lesions were submitted for cytological, histophatological, microbiological and molecular analysis, and confirmed the diagnosis of *Nocardia africana*.

## Discussion

Nocardiosis is an unusual clinical disease in companion animals. In cats, cutaneous-subcutaneous lesions are the main clinical sign. Usually, cutaneous-subcutaneous signs include abscesses, pustules, fistulas, cellulitis, and mycetoma, generally associated with draining of purulent secretion containing granules [12]. Our cat also presented cellulitis with draining sinuses containing granules, reinforcing the skin lesions as the most common clinical manifestation of feline nocardiosis [4].

Less frequently feline nocardiosis cause pulmonary and systemic signs [3]. Complications from cutaneous-subcutaneous nocardiosis in companion animals are represented by invasion of adjacent structures, leading to pulmonary, neurologic and/or disseminated (systemic) forms [1,4]. However, rarely feline nocardiosis have been associated with osteomyelitis [13]. In our cat was diagnosed uncommon mandibular osteomyelitis with intense bone proliferation and osteolysis. The transmission of microorganism in cats is associated with inhalation of soil organisms, consumption of contaminated foods, and inoculation through wounds, such as caused by territorial fights with other felids [1,14]. Despite no history of previous traumatic lesion in mandibular region or mouth in our cat, the ostemyelitis caused by *Nocardia africana* probably occurred secondary to periodontitis, due to ingestion of contaminated foods, or traumatic wounds in the gingiva, resembling the transmission route of nocardiosis and actinomycois in livestock [15].

Clinical laboratory findings in feline nocardiosis are usually nonspecific [3]. In the present report was observed leukocytosis and monocytosis, which are hematologic abnormalities observed in feline nocardiosis [3,5]. Severe osteolysis observed in radiographic image of mandibula in our cat was indicative of guarded prognosis, although bone infections rarely have been observed in feline nocardiosis [3,13].

Traditionally, *Nocardia* species isolated from companion animals have been distinguished by phenotypic characteristics, based on microbiological culture, biochemical characterization, carbohydrate hydrolysis, and antimicrobial susceptibility patterns [5]. In the current report, the microorganism was isolated from samples obtained from a lesion after 72 h of incubation on both sheep blood agar and Sabouraud agar, reinforcing the importance of microbiological culture as a valuable method in the routine diagnosis of *Nocardia*.

In the present report, the isolate was initially identified as belonging to the *N. nova* complex [7]. Expanded biochemical test and antibiotic susceptibility patterns were used to differentiate members of *Nocardia nova* complex. Gram, Panoptico, and modified Ziehl-Neelsen stains revealed the presence of gram-positive, acid-fast, branching filamentous organisms suggestive of *Nocardia* sp. The histologic findings revealed a suppurative necrosis and abscesses formation usually observed in feline nocardiosis [3,12]. Our data highlighted the importance of combination of clinical and epidemiological data, hematologic abnormalities, radiographic images, microbiological culture, cytological and histopathological examination to improve the diagnosis of feline nocardiosis.

Sulfonamide-trimethoprim, aminoglicosides and late generation β-lactam drugs are the choices in therapy of feline nocardiosis. Successful treatment of cats with nocardiosis has been observed mainly in skin lesions [3,12]. In contrast, severe complications and death have been related with disseminated forms (systemic), particularly in cats co-infected with immnunosuppressive diseases [4,16]. In our cat, despite negative results for feline immunosuppressive diseases, therapy based on antimicrobial susceptibility testing and supportive care, the animal showed progressive worsening of body condition and died.

Recently, polymerase chain reaction, restriction fragment length polymorphisms, DNA- hybridization, and gene sequencing have enabled reclassification of some species of *Nocardia*. *Nocardia cyriacigeorgica*, *Nocardia brasiliensis, Nocardia otitidiscaviarum*, and *Nocardia nova* complex have been the most common species involved in feline nocardiosis [3]. *Nocardia nova* complex comprises *Nocardia africana*, *Nocardia nova*, *Nocardia veterana*, and *Nocardia kruczakiae* species based in recent molecular classification [17]*. Nocardia africana* infections are considered to be rare in companion animals. *Nocardia africana* was reported in feline mycetoma in Japan [18]. In addition, 17 cases of feline nocardiosis were described recently in Australia with involvement of *Nocardia nova* in cutaneous and systemic infections [4], although these cases have been diagnosed using phenotypic methods.

Currently, human nocardiosis has emerged as an increasingly common opportunistic pathogen among immunosuppressed patients, particularly those infected by AIDS, and other debilitating diseases [19]. Clinically, cutaneous-subcutaneous, pulmonary and/or neurological signs constitute the most common clinical manifestations of human nocardiosis [1]. *N. asteroides*, *N. brasiliensis*, *N. farcinica* and *N. nova* are species most frequently associated to human disease [19]. The species of *Nocardia* associated with human nocardiosis are similar to those most frequently found in domestic animals [5].

The main route of transmission of the genus *Nocardia* for people appears to be inhalation of the organism in dry warm climate regions, that facilitate the aerosolization and dispersal of pathogen, or caused by traumatic skin inoculation of organism from soil [1,3,19]. Microorganisms of the genus *Nocardia* probably are not transmitted from person-to-person form [19], and the impact of infected domestic animals in transmission of the disease to humans remains unclear [3], although cutaneous-subcutaneous infections transmitted to people by bite or scratch wounds from cats have been described elsewhere [20,21]. Thus, precautions should be taken by humans affected by immune dysfunction, with special attention to avoid contact with soil or organic matter contaminated by domestic animals, and management of animals suspected of nocardiosis, particularly companion animals due to close contact with humans.

## Conclusion

The present report describes a rare clinical case of feline osteomyelitis caused by *Nocardia africana*, diagnosed based upon a combination of clinical signs, microbiological culture, cytological and histopathological findings, radiographic images, and molecular methods. The use of modern molecular techniques constitutes a valuable method for *Nocardia* species identification, and may contribute to identification to new species of *Nocardia* that are virulent in cats.

The study was approved by Ethic Commitee of College of Veterinary Medicine, Pontifícia Universidade Católica, São José dos Pinhais, State of Paraná, Brazil.

## Competing interests

The authors declare that there are no conflicts of interest in this study.

## Authors’ contributions

The manuscript was prepared by MGR, MRF, and LAZC, and critically discussed by other authors. Clinical examination, diagnosis and treatment of animal were performed by MRF, JW, SMR, CZC. Initial microbiological culture and characterization of the genus *Nocardia* was performed by KDC. Phenotypic identification and storage of isolate was carried out by LAZC and MGR. Molecular analysis of isolate was carried out by TG, TM, and KY. All authors read and approved the final manuscript.

## References

[B1] Beaman BL Beaman L *Nocardia* species: host-parasite relationships Clin Microbiol Rev 1994 7 213 264 8055469 10.1128/cmr.7.2.213 PMC358319

[B2] Quinn PJ Carter ME Markey B Carter GR Clinical Veterinary Microbiology 1994 1994 London: Wolfe

[B3] Greene CE Infectious diseases of the dog and cat 2012 4 St. Louis, Missouri: Elsevier

[B4] Malik R Krockenberger MB O`Brien CR White JD Foster D Tisdall PLC Gunew M Carr PD Bodell L McCowan C Howe J Oakley C Griffin C Wigney DI Martin P Norris J Hunt G Mitchell DH Gilpin C *Nocardia* infections in cats: a retrospective multi-institutional study of 17 cases Australian Veterinary J 2006 84 235 245 10.1111/j.1751-0813.2006.00004.x 16879126

[B5] Ribeiro MG Salerno T Mattos–Guaraldi AL Camello TCF Langoni H Siqueira AK Paes AC Fernandes MC Lara GHB Nocardiosis: an overview and additional report of 28 cases in cattle and dogs Revista do Instituto de Medicina Tropical 2008 50 177 185 10.1590/S0036-46652008005000004 18516465

[B6] Kiska DL Hicks K Pettit DJ Identification of medically relevant *Nocardia* species with an abbreviated battery of test J Clinical Microbiology 2002 40 1346 1351 10.1128/JCM.40.4.1346-1351.2002 PMC140358 11923355

[B7] Murray PR Baron EJ Jorgensen JH Pfaller MA Yolken RH Manual of Clinical Microbiology 2003 Eight Washington: AMS Press

[B8] Kageyama A Torikoe N Yazawa K Mikami Y Nishimura K *Nocardia asiatica* sp. nov. pathogen isolated from patients with nocardiosis in Japan and clinical specimes from Thailand Int J Syst Evol Microbiol 2004 54 123 130 10.1099/ijs.0.02676-0 14742469

[B9] Saitou N Nei M The neighbor-joining method: a new method for reconstructing phylogenetic trees Mol Biol Evol 1987 4 406 425 3447015 10.1093/oxfordjournals.molbev.a040454

[B10] Takamura K Dudley J Nei M Kumar S MEGA4: Molecular Evolutionary Genetics Analysis (MEGA) software version 4.0 Mol Biol Evol 2007 24 1596 1599 10.1093/molbev/msm092 17488738

[B11] Clinical and Laboratory Standards Institute Performance standards for antimicrobial disk susceptibility tests. Approved standard 2006 Eighth Wayne, USA: CLSI document M2-A9

[B12] Scott W Miller WH Griffin CE Small Animal Dermatology 2001 6 Philadelphia: WB Saunders

[B13] Bradney IW Vertebral osteomyelitis due to *Nocardia* in a dog Aust Vet J 1985 62 315 316 10.1111/j.1751-0813.1985.tb14914.x 4074219

[B14] Luque R Astorga C Tarradas B *Nocardia otitidiscaviarum* infection in a cat Vet Rec 2002 151 488 12418540

[B15] Radostits OM Blood DC Gay CC Veterinary Medicine – A Textbook of the Diseases of Cattle, Sheep, Pigs, Goats and Horses 2007 Philadelphia: Baillière Tindall

[B16] Davenport DJ Johnson GC Cutaneous nocardiosis in a cat J Am Vet Med Assoc 1986 188 728 729 3700231

[B17] Brown-Elliott BA Brown JM Conville PS Wallace RJ Clinical and laboratory features of the *Nocardia* spp. based on current molecular taxonomy Clin Microbiol Rev 2006 19 259 282 10.1128/CMR.19.2.259-282.2006 16614249 PMC1471991

[B18] Hattori Y Kano R Kunitani Y Yanay T Hasegawa A *Nocardia africana* isolated from a feline mycetoma J Clin Microbiol 2003 41 908 910 10.1128/JCM.41.2.908-910.2003 12574314 PMC149653

[B19] Corti ME Villafañe-Fioti MF Nocardiosis: a review Int J Infect Dis 2003 7 243 250 10.1016/S1201-9712(03)90102-0 14656414

[B20] Sachs MK Lymphocutaneous *Nocardia brasiliensis* infection acquired from a cat scratch: case report and review Clin Infect Dis 1992 15 710 711 10.1093/clind/15.4.710 1420688

[B21] Bottei E Flaherty JP Kaplan LJ Duffee-Kerr L Lymphocutaneous *Nocardia brasiliensis* infection via cat scratch: a second case Clin Infect Dis 1994 18 649 650 8038327 10.1093/clinids/18.4.649

